# Heart rate and electrodermal activity during key driving events in older adult drivers with and without elevated amyloid burden

**DOI:** 10.1002/alz.089686

**Published:** 2025-01-03

**Authors:** Anu M Kumar, Yi Lu Murphy, Amanda Cook Maher, Carol C Persad, Jordan R Bross, Emma Flynn, Robert A. Koeppe, Bruno Giordani

**Affiliations:** ^1^ University of Michigan, Ann Arbor, MI USA; ^2^ University of Michigan ‐ Dearborn, Dearborn, MI USA; ^3^ University of Michigan Medical School, Ann Arbor, MI USA

## Abstract

**Background:**

Early symptoms of Alzheimer’s disease (AD) may include subtle performance changes in complex tasks, such as driving, which could be potential markers for identifying those at risk of cognitive decline. In this study, categorical driving behaviors and events as well as related physiological changes were compared in older adults with and without elevated brain amyloid.

**Method:**

Video and physiological data collected from 21 amyloid positive and 21 amyloid negative participants over the age of 65 (range 65‐85), who participated in the University of Michigan’s Driving and Physiological Responses study were analyzed. Amyloid positivity was determined based on the PET centiloid scale. All drivers completed the same fixed course route. Road‐ and driver‐view videos of each participant were annotated to mark 69 key road events and driving behaviors (e.g., if the participant made a rolling or full stop). Heart rate (HR) and electrodermal activity (EDA) data collected through an Empatica E4 watch were obtained for each road event and categorized as “high” or “low”. Physiological responses were considered “high” if they exceeded the driver’s average physiological response during the fixed course drive and “low” if they did not. Chi‐square analyses were used with a p‐value of ≤ 0.05.

**Result:**

Older adults with high amyloid burden exhibited a higher frequency of rolling through stop signs compared to their amyloid negative peers and exhibited “high” HR responses during this event. Amyloid positive drivers were also more likely to exhibit a “high” HR at intersections without traffic signs and when encountering medium/high traffic loads. No significant patterns in EDA were found.

**Conclusion:**

Amyloid positive older drivers exhibited relatively elevated HR responses while driving in more complex situations (at stop signs or heavier traffic). This pattern of driving behavior and accompanying physiological responses that account for participants’ individual variability may indicate an early sign of cognitive change in older individuals with high amyloid burden. These attributes can be used as effective features to build a Machine Learning‐based AD diagnosis tool to detect early‐stage AD in older drivers.

